# Upward shift of the vortex solid phase in high-temperature-superconducting wires through high density nanoparticle addition

**DOI:** 10.1038/srep20436

**Published:** 2016-02-08

**Authors:** Masashi Miura, Boris Maiorov, Fedor F. Balakirev, Takeharu Kato, Michio Sato, Yuji Takagi, Teruo Izumi, Leonardo Civale

**Affiliations:** 1 Materials Physics and Applications Division, Los Alamos National Laboratory, Los Alamos, New Mexico 87545, USA; 2 Graduate School of Science & Technology, Seikei University, 3-3-1 Kichijoji-kitamachi, Musashino-shi, Tokyo 180-8633, Japan; 3 Materials R&D Laboratory, Japan Fine Ceramics Center, 2-4-1 Mutsuno, Atuta-ku, Nagoya 456-8587, Japan; 4 Superconductivity Research Laboratory, International Superconductivity Technology Center, KSP R&D Wing A-9F, 3-2-1 Sakado, Takatsu-ku, Kaswasaki-shi, Kanagawa, 213-0012 Japan

## Abstract

We show a simple and effective way to improve the vortex irreversibility line up to very high magnetic fields (60T) by increasing the density of second phase BaZrO_3_ nanoparticles. (Y_0.77_,Gd_0.23_)Ba_2_Cu_3_O_*y*_ films were grown on metal substrates with different concentration of BaZrO_3_ nanoparticles by the metal organic deposition method. We find that upon increase of the BaZrO_3_ concentration, the nanoparticle size remains constant but the twin-boundary density increases. Up to the highest nanoparticle concentration (*n* ~ 1.3 × 10^22^/m^3^), the irreversibility field (*H*_irr_) continues to increase with no sign of saturation up to 60 T, although the vortices vastly outnumber pinning centers. We find extremely high *H*_irr_, namely *H*_irr_ = 30 T (**H**||45°) and 24 T (**H**||*c*) at 65 K and 58 T (H||45°) and 45 T (**H**||*c*) at 50K. The difference in pinning landscape shifts the vortex solid-liquid transition upwards, increasing the vortex region useful for power applications, while keeping the upper critical field, critical temperature and electronic mass anisotropy unchanged.

Although the critical temperature (*T*_c_) and upper critical field (*H*_c2_) of high temperature superconductors are extremely high, superconductors only become technically useful at a much lower field-temperature boundary, called the melting, or irreversibility, line. This is due to the appearance of the vortex liquid phase, one of the unique characteristics of these superconductors. In this liquid phase, superconductors have finite resistivity behaving much like a normal metal, thus the need to move the melting line to higher fields/temperatures. Defects can move the melting line, with random point-like defects^1^ pushing it down and correlated defects moving this line up. The positive effect of correlated disorder is restricted to an angular range near the defects’ orientation, and usually only up to a characteristic field related to the density of the defects (the matching field, typically a few tesla)^2^,^3^,^4^,^5^. Pushing up this upper limit is both technologically and scientifically important; given the interest in high magnetic field magnets. This requires finding ways to immobilize vortex densities much higher than the highest defect density that can be introduced without compromising the material integrity.

Recently, a third kind of disorder (neither correlated nor point-like) has been demonstrated to be effective at improving both *J*_c_ and *H*_irr_ at a very wide range of field orientations^6^,^7^,^8^,^9^,^10^,^11^,^12^,^13^,^14^. A deeper and more quantitative understanding of the effect of nanoparticles in a mixed pinning landscape (as they often work together with correlated defects) is necessary for assessing the potential of Cu-based superconducting wires for applications at high fields^15^.

In particular, it is important to establish the behavior of *H*_irr_ when the vortex density is much higher than that of the defects. Most REBa_2_Cu_3_O_*y*_ (REBCO) films (RE = rare earth) with self-assembled nanorods^4^,^5^ and YBCO crystals with columnar defects^2^,^3^ show improved *H*_irr_ and a “kink” in the vortex liquid-glass transition for **H**||*c*. This occurs when vortices stop populating columnar defects and start filling interstitial positions, at a density related to that of the defects at the so-called matching field (μ_0_*H*_ϕ_). These findings lead to the validation of the simple argument that once the pinning centers have been outnumbered by vortices, their effect on increasing *H*_irr_ decreases or may even be washed out. However, this idea overlooks the fact that vortices are interacting elastic objects, which form a lattice with different degrees of order depending on the underlying pinning landscape. The understanding of the physical system adds a layer of complexity when pinning centers of different dimensions (point defects, lines, planes or nanoparticles) are taken into account.

In this paper, we show that the combination of planar twin boundaries (TBs) and a higher density of three dimensional (3D) nanoparticles (NPs) increases *H*_irr_ at up to 60 T. In the case of TBs there is a clear signature of the matching field at very low fields (0.5 T) but nevertheless, their presence continues to increase *H*_irr_ up to 60 T. The effect of the NPs is also seen up to 60 T with the additional benefit of their being effective in the entire angular range. We find no signs of saturation in the improvement of *H*_irr_ up to 3 wt% BaZrO_3_ (BZO) added to REBCO wire up to the highest field measured (60 T), and obtain record high values.

## Results

The results shown are distributed as follows. We start by explaining the growth methods and show superconducting and microstructural studies. We find that the use of (Y,Gd)BCO is beneficial (as compared to YBCO) and that the addition of BZO NPs does not degrade the general properties, and increases twin boundary density. Then we focus on transport measurements in DC and pulsed fields as a function of temperature and field orientation. For the upper critical field, (*H*_c2_), the addition of NPs produces no significant changes. For *H*_irr_, the scenario is complex and depends on the field magnitude and orientation. For **H**||*c*, at low fields, twin boundaries dominate the irreversibility line with a matching field observed and a peak as function of field orientation. As the field increases the effects of TBs are less prominent as nanoparticles compete in the vortex localization. At high fields, NPs and TBs cooperate to enhance the *H*_irr_. At intermediate angles (e.g. **H**||45°) nanoparticles produce the greatest increase in *H*_irr_, showing no saturation up to the highest fields measured. Furthermore, at high fields we obtain a constant improvement of 25% with respect to YBCO films. For **H**|| *ab*, the changes due to NPs addition are small as compared to other orientations.

### Uniformly dispersed BaZrO_3_ nanoparticles in (Y_0.77_Gd_0.23_)Ba_2_Cu_3_O_*y*_ wires

**Figure 1:**
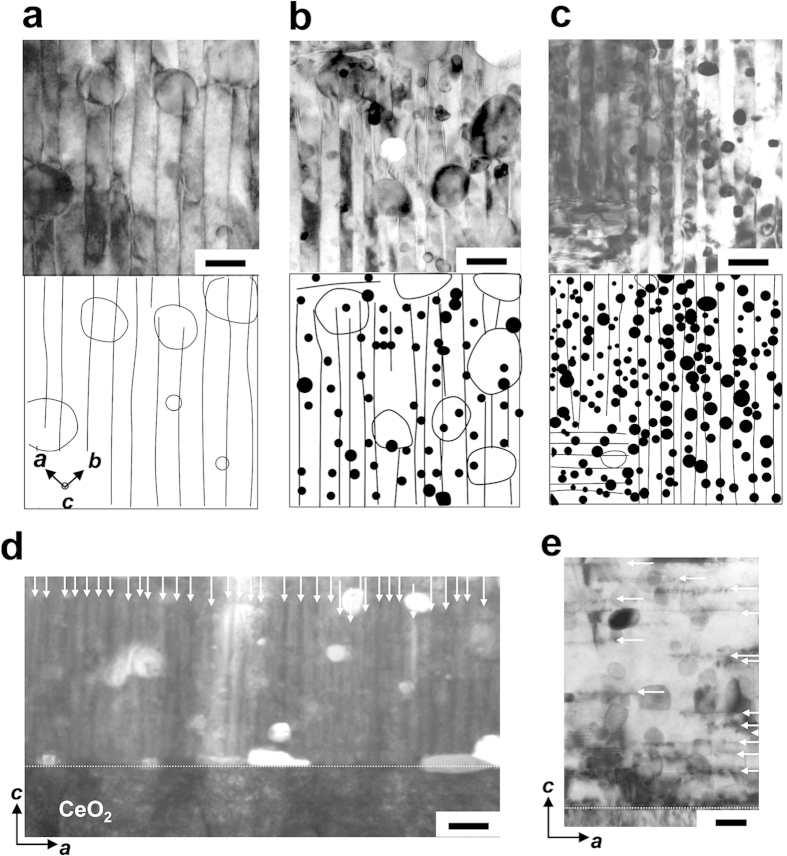
Microstructural characterization for YBCO and (Y,Gd)BCO+BZO wires. Top panels of (**a–c**): High magnification planar view TEM images of (**a**) YBCO, (**b**) +1BZO and (**c**) +3BZO wires, respectively. Bottom row: corresponding positions of TBs and BZO NPs. In the bottom panels, some of the RE_2_Cu_2_O_5_ precipitates are indicated by open circles. (**d**) Wide areas of cross-section TEM image of +3BZO wires. The TBs are indicated by arrows. (**e**) Cross-section high-magnification TEM image of +3BZO wire showing randomly dispersed BZO NPs and a high density of SFs (white arrows). Horizontal bars in Fig. 1 (a,b,c,d) and Fig. 1 (e) represent 100 nm and 30 nm, respectively.

The samples used in this study were 0.5 μ thick films of standard YBCO, (Y_0.77_Gd_0.23_)Ba_2_Cu_3_O_*y*_ ((Y,Gd)BCO) and BZO-doped (Y,Gd)BCO ((Y,Gd)BCO+BZO) derived from the metal organic deposition (MOD) process, grown on metal substrates. The content of BZO in the films was 1–3 wt%. The film microstructure was studied by several techniques (see Methods). The planar view transmission electron microscopy (TEM) images of standard YBCO, (Y,Gd)BCO with 1wt% BZO (+1BZO) and 3wt% BZO (+3BZO) wires are shown in Fig. 1a–c, respectively. For clarity, the corresponding bottom panels in those figures show schematics of the main defects. All films have a low density (∼0.1 × 10^21^/m^3^) of large RE_2_Cu_2_O_5_ (225) precipitates (shown as open circles in the bottom panels) formed during REBCO crystallization from the precursors containing BaF_2_, RE_2_Cu_2_O_5_ and CuO. In both +1BZO and +3BZO wires, BZO NPs (modal size ∼23 nm) are randomly distributed and uniformly dispersed. The average density of these nanoparticles was determined to be *n* = 2.9 × 10^21^/m^3^ for +1BZO, *n* = 7.1 × 10^21^/m^3^ for +2BZO (not shown), and *n* = 13 × 10^21^/m^3^ for +3BZO wires, respectively. Note that the greater than linear dependence of *n* on BZO% derives from the slight decrease in size of the NPs with increasing BZO density. In addition to the uniformly randomly distributed 3D defects (BZO NPs), all samples, including the standard YBCO, contain high densities of two types of planar (2D) defects, namely *c*-axis correlated TBs (Fig. 1a–d) and stacking faults (SFs) parallel to the *ab* plane (Fig. 1e). In some previous studies of MOD REBCO with nanoparticles, it was observed that the SFs cut into the TBs, resulting in discontinuous short length TBs^14^. In contrast, although our +3BZO wire has a high density of SFs as shown in Fig. 1e, the TBs remain connected from the bottom to the surface (Fig. 1d), indicating that the TBs in the BZO NP-doped wire are *c*-axis correlated pinning centers.

Table 1 shows a summary of the crystallographic and superconducting properties for the YBCO, (Y,Gd)BCO, +1BZO, +2BZO and +3BZO films. No decrease in *T*_c_, or increase of *δω* or *δϕ* is observed for the films with BZO inclusions. The only difference found is positive, being that films with Y,Gd have higher *T*_c_ and *self-field J*_c_(*J*_c_^s.f.^) than the YBCO film, consistent with good crystallinity (unchanged *δω, δϕ*). The *J*_c_^s.f.^ values of the +3BZO film at 65 and 77 K are 12.5 and 4.0 MA cm^−2^, respectively. These *J*_c_^s.f.^ values are almost as high as those of nanocomposite MOD-YBCO thin films on a single crystal substrate^6^. The fact that we maintained (or even improved) *J*_c_^s.f.^ and *T*_c_ after adding defects is significant given that in some cases, for films grown by pulsed laser deposition (PLD), *T*_c_ and *J*_c_^s.f^ decreased with increasing BZO content due to poor crystallinity, local strain and oxygen deficiency near second phases^16^. The difference in *T*_*c*_ and *J*_c_^s.f^ behavior with BZO% between MOD and PLD processes comes from differences in the formation mechanisms of the BZO/REBCO matrix^17^.

### Irreversibility line at low and intermediate DC magnetic fields

**Figure 2:**
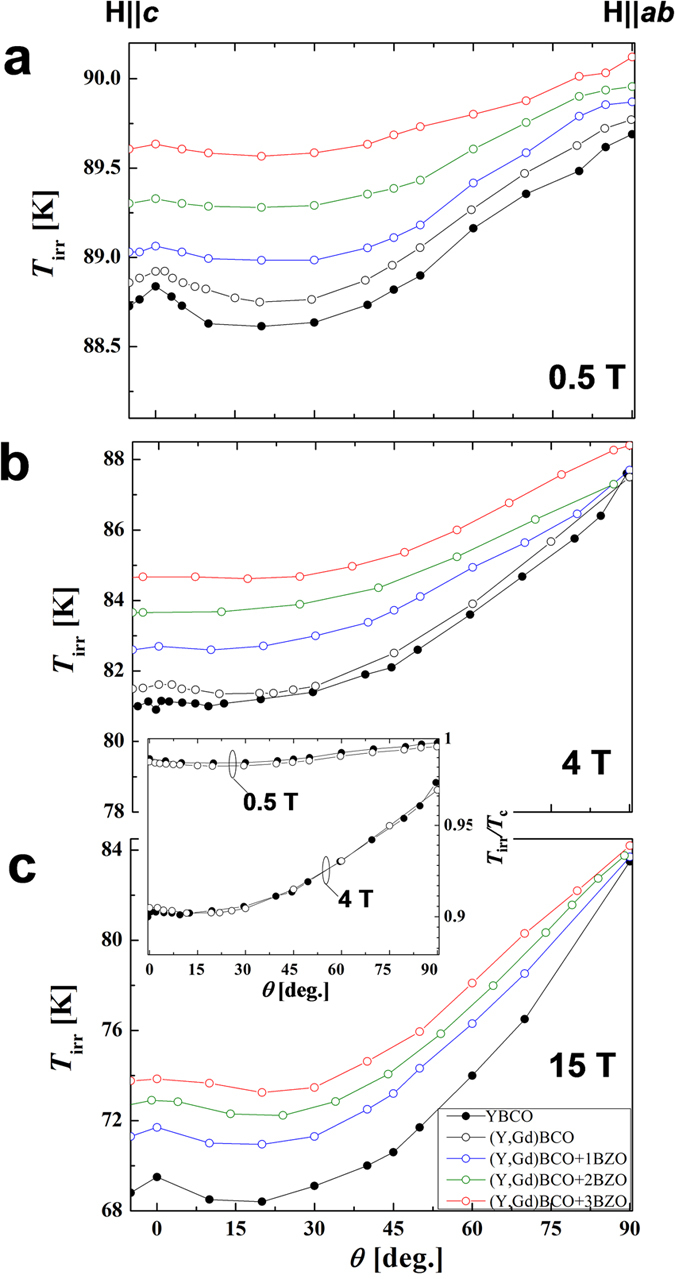
Angular dependent irreversibility temperature *T*_irr_. Angular dependence of *T*_irr_ for YBCO, (Y,Gd)BCO and (Y,Gd)BCO+BZO wires at (**a**) 0.5 T, (**b**) 4 T and (**c**) 15 T, respectively. Inset of Fig. 2 indicates the angular dependent normalized *T*_irr_ (*T*_irr_/*T*_c_) for YBCO and (Y,Gd)BCO wires.

To investigate the influence of the density of BZO NPs on the superconducting properties of the glassy phase, we measured the irreversibility temperature *T*_irr_ (*H*,*θ*). Figure 2a–c show *T*_irr_ (*θ*) at 0.5, 4 and 15 T for standard YBCO, (Y,Gd)BCO and (Y,Gd)BCO+BZO wires, where *θ* is the angle between the magnetic field and the *c* axis.

For the YBCO and (Y,Gd)BCO wires, at low magnetic fields we observe two maxima in *T*_irr_, centered at **H**||*c* (*θ* = 0°) and **H**||*ab* (*θ* = 90°). The peak at **H**||*c* comes from *c*-axis correlated defects. The maximum at **H**||*ab* originates from the electronic-mass anisotropy and the SFs. At intermediate fields (see Fig. 2b), the *c*-axis peak of *T*_irr_ in both YBCO and (Y,Gd)BCO becomes very weak, but is evident again at higher fields (see Fig. 2c). We note that the *T*_irr_(θ)/*T*_c_ for the YBCO and (Y,Gd)BCO wires are almost identical as shown in inset of Fig. 2, which validates the use of YBCO for the comparison that we are making.

All wires with BZO NPs show an enhanced *T*_irr_(*θ*) as compared to that of the YBCO and (Y,Gd)BCO wires, and the magnitude of the *T*_irr_(*θ*) enhancement increases with increasing BZO NP content at all magnetic fields (0.5, 4 and 15 T) and at all angles. The *T*_irr_ anisotropy, *T*_irr_(||*ab*)/*T*_irr_(||*c*), is also smaller for the +BZO samples and decreases monotonically with BZO NP content. At low fields, the +3BZO wire exhibits nearly isotropic properties with a small (in height) *c*-axis peak. At 15 T, *T*_irr_ (*θ*) shows a small *c*-axis peak indicating a contribution from correlated pinning. This is consistent with *J*_c_(*θ*) data^10^ at high fields where a *c*-axis peak is also found. In both cases (*J*_c_ and *T*_irr_) the *c*-axis peak height is less pronounced in samples with BZO additions, partially because of the general increase in *J*_c_ and *T*_irr_ produced by the nanoparticles.

**Figure 3:**
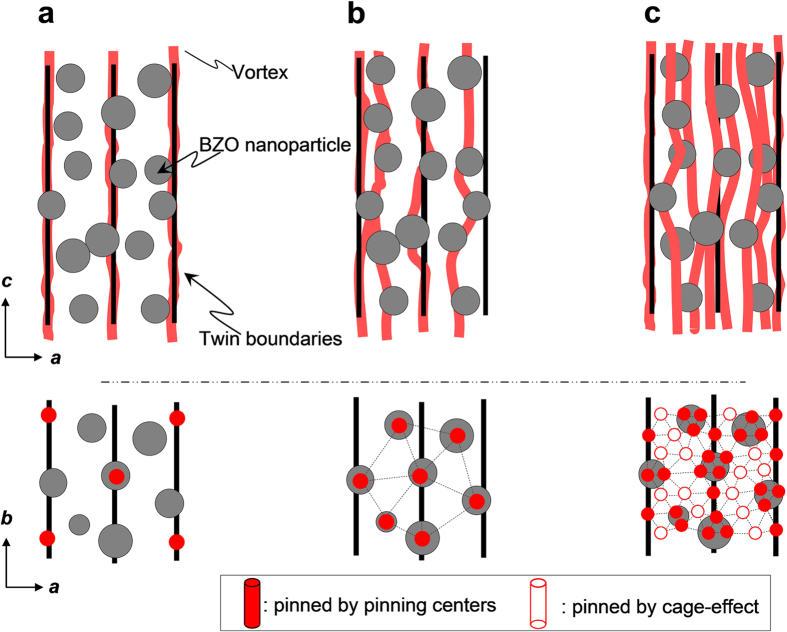
Diagrams of vortex pinning for configurations involving different defects at H||c. (**a**) Diagram of vortex and *c*-axis parallel correlated TBs and BZO NPs at μ_0_*H*<μ_0_*H*_ϕ,TB_. (**b**) Diagram of a vortex and BZO NPs and TBs at μ_0_*H* ∼ μ_0_*H*_BZO_(*a*_f_ = *d*_BZO_), showing that above μ_0_*H*_ϕ,TB_ vortices are localized not only by TBs but also by BZO NPs. (**c**) Diagram of a vortex and hybrid defects at μ_0_*H* ≫ μ_0_*H*_BZO_, showing how a large number of vortices are arrested by BZO NPs and TBs.

The results in Fig. 2 can be interpreted as follows. At low fields, below the matching field of the TBs (μ_0_*H*_*ϕ,*TB_) given by μ_0_*H*_*ϕ,*TB_ = (*ϕ*_0_/*d*_TB_^2^), where *ϕ*_0_ and *d*_TB_ are the flux quantum and TB spacing respectively, most vortices get localized by the TBs (Fig. 3a). At intermediate *H,* above μ_0_*H*_*ϕ,*TB_, the density of vortices is high enough that the TBs become “saturated”, the extra vortices sit outside them and become localized by the randomly distributed NPs which are strong pinning centers (Figs 2b and 3b). At even higher fields, once all the NPs are saturated, no elastic energy is lost by achieving pinning at the TBs, allowing for the localization effect of the TBs to be observed through the caging effect (Fig. 3c), similar to what happens for the standard YBCO sample (Fig. 2c).

**Figure 4:**
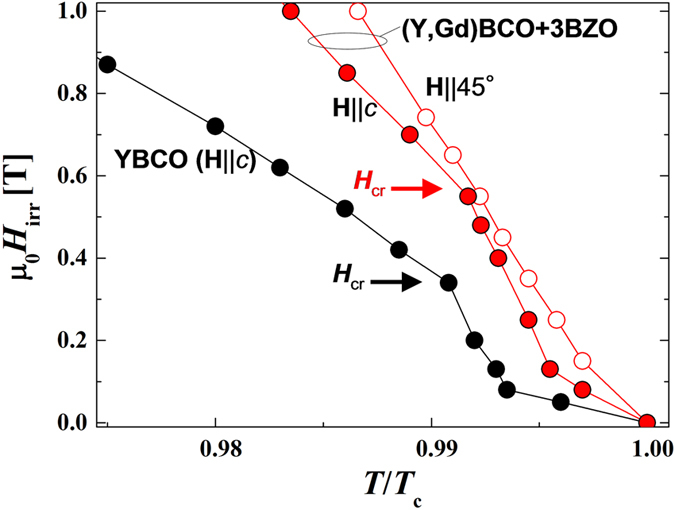
Temperature dependence of *H*_irr_ at low magnetic fields. Red open and solid symbol show μ_0_*H*_irr_(*T*/*T*_c_) at **H**||45° and **H**||*c*, respectively, for the +3BZO wire. Black solid symbols indicate μ_0_*H*_irr_(*T*/*T*_c_) for the YBCO wire at **H**||*c*. A clear kink (*H*_cr_) is observed only for **H**||*c* for the wires.

**Figure 5:**
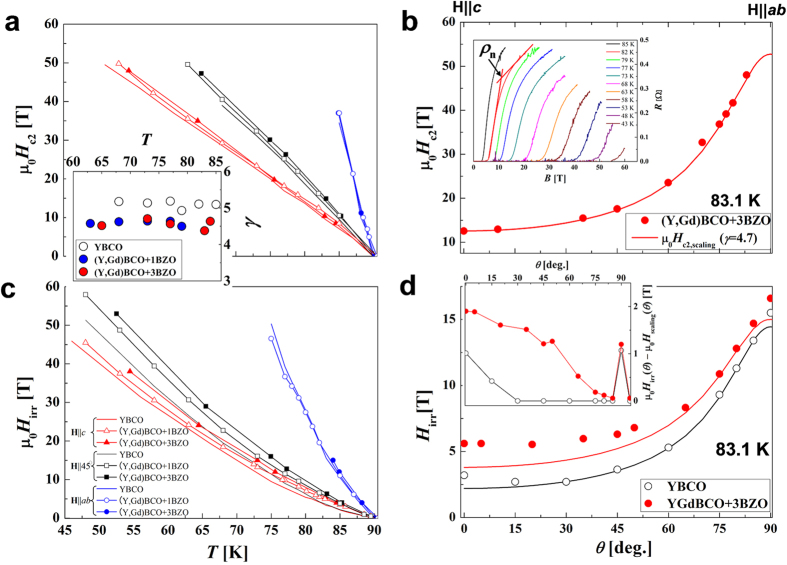
Temperature and angular dependence of the upper critical field and irreversibility field. (**a**) Temperature and (**b**) angular dependence of μ_0_*H*_c2._ The inset of Fig. 5(a) shows the temperature dependence of *γ* calculated using 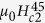 and 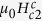. The inset of Fig. 5(b) shows *ρ*_n_(*T*) with **H**||*c* for YGdBCO + 1BZO wire. Solid lines in Fig. 5(b) are *ε*(*θ*)μ_0_*H*_c2_ using *γ* values from the inset of Fig. 5(a). (**c**) Temperature and (d) angular dependence of μ_0_*H*_irr_. Solid lines in Fig. 5(d) are *ε*(*θ*)μ_0_*H*_irr_ for the YBCO and +3BZO wires using *γ* = 5.1 and 4.7, respectively.

To better determine the effects of the *c*-axis correlated defects, we measured μ_0_*H*_irr_(*T*) at low fields for different *θ* (see Fig. 4). The *c*-axis irreversibility lines of the wires with and without BZO NPs exhibit a pronounced kink indicating a crossover field (see *H*_cr_ arrows). For fields smaller than μ_0_*H*_cr_, *H*_irr_ shows a rapid increase with decreasing *T*; above μ_0_*H*_cr_ the irreversibility line is closer to a linear temperature dependence. For the +3BZO wire, μ_0_*H*_cr_ is 0.55 T, which is 1.6 times higher than that of YBCO (μ_0_*H*_cr_ = 0.34 T). From TEM images, we observe that this correlates with the higher TB density found in +3BZO as compared to YBCO wires. Indeed, we obtain μ_*0*_*H*_*ϕ*,TB_ = 2.3T and 1.0T, for +3BZO and YBCO, respectively. This cross-over is remarkably similar to that observed in YBCO bulk with nanoscale TBs^18^,^19^ and columnar defects^20^. For TBs and columnar defects, the cross over field is usually not observed precisely at the matching field but around 1/3-1/2 of *H*_*ϕ*_. This also correlates very well with our observation of the crossover fields with *H*_cr_/*H*_ϕ,TB_ ∼ 1/3 for samples with and without BZO NPs, namely 0.34 and 0.24 for YBCO and +3BZO, respectively. To further corroborate that the observed crossover field arises from correlated defects and is not an artifact, we confirmed that no kink is found in *H*_irr_-*T* at **H**||45° (see Fig. 4). Therefore, we can conclude that the array of TBs is the main source of correlated pinning at **H**||*c* in our MOD REBCO wires. Note that the TBs continue to contribute at μ_0_*H* ≫ μ_0_*H*_cr_ as seen in the peak of *T*_irr_(*θ*) around **H**||*c* in Fig. 2c and the inset of Fig. 5d.

### Irreversibility lines and upper critical fields in pulsed fields

To obtain the temperature and angular dependence of μ_0_*H*_c2_ and μ_0_*H*_irr_ for the YBCO and (Y,Gd)BCO + BZO wires at even higher fields, we measured *ρ*(*T*) and *ρ*(*H*) at various *θ* values by using pulsed fields up to 60 T (see insert Fig. 5b). See Methods for further details.

Figure 5a shows μ_0_*H*_c2_(*T*) at **H**||*c*, **H**||45°, and **H**||*ab*. The results demonstrate that μ_0_*H*_c2_(*T*) is remarkably similar for all samples in spite of their very different pinning landscapes. The inset of Fig. 5a shows the electronic-mass anisotropy (*γ*) vs. *T* obtained from the *H*_c2_ measurements for the YBCO and (Y,Gd)BCO+BZO wires (see ref. 9 for details). The average values for *γ* are 5.1, 4.6 and 4.7 for the YBCO, +1BZO and +3BZO wires, respectively. Although *γ* is slightly smaller for the wires with BZO, all values are very close to 5. Further confirmation of the obtained value of *γ*, comes from the angular-dependent *H*_c2_ shown in Fig. 5b. The angular dependence of *H*_c2_ can be fit very well using *Hε*(*θ*) = *H*(cos^2^(*θ*)+*γ*^-2^sin^2^(*θ*)) (see ref. 21) with *γ* = 4.7, in agreement with the experimental result shown in the inset of Fig. 5a. This is also in accordance with our previous work, where we reported that the μ_0_*H*_c2_(*θ*) dependence follows a single curve consistent with *γ* ~ 5 even with different additions and growth methods^22^. From the μ_0_*H*_c2_ data, we conclude that μ_0_*H*_c2_ is not greatly affected by the pinning landscape. It is worth noting that the value of *γ* ~ 5 is indeed smaller than the values found for YBCO single crystals^23^,^24^, as previously noted^25^. The lack of sensitivity in *H*_c2_ upon increase of disorder can be found in the small coherence length of REBCO (also the reason for high *H*_c2_). The small coherence length places REBCO in the clean limit, thus making its superconducting properties less sensitive to disorder.

On the other hand, the changes in pinning landscape do influence *H*_irr_, as a clear enhancement is found for all +BZO wires compared with YBCO wire (see Fig. 5c,d). Figure 5c displays the μ_0_*H*_irr_-*T* at various angles for the YBCO and (Y,Gd)BCO+BZO wires. For **H**||*c* and **H**||45°, wires with BZO NPs show a higher μ_0_*H*_irr_(*T*) than that of standard YBCO. Furthermore, we observe that μ_0_*H*_irr_(*T*) continues to increases with higher densities of BZO NPs. For **H**||*ab*, +BZO samples show a much smaller increase in μ_0_*H*_irr_(*T*).

The changes can be better understood by analyzing the *H*_irr_(*θ*) as shown in Fig. 5d. Consistent with Fig. 5c, the addition of BZO NPs produces a small increase near **H**||*ab* and a much bigger enhancement for **H**||*c* and intermediate orientations. The resulting angular dependence of *H*_irr_ for the +3BZO wire cannot be described using the electronic-mass anisotropy scaling model, unlike *H*_c2_(*θ*) or YBCO’s *H*_irr_(*θ*), which do follow *ε*(*θ*) for most of its angular dependence (see Fig. 5b,d). More information on the effect of the nanoparticles on *H*_irr_ can be observed in the inset to Fig. 5d. In this inset we plot the difference of μ_0_*H*_irr_(*θ*)–*ε*(*θ*)μ_0_*H*_irr_ for the YBCO and +3BZO sample. For YBCO we observe that, except for the peaks around **H**||*c* and **H**||*ab* due to correlated disorder (TBs and SFs, respectively), over most of the angular range *H*_irr_(*θ*) can be very well described by the electronic-mass anisotropy, *γ* = 5 indicating that most of the angular dependence of *H*_irr_ can be explained by pinning arising from weak random point defects such as oxygen vacancies (with diameter in the angstrom scale ≪ 2ξ(*T*)). For +3BZO we find very similar behavior near the *ab*-plane, where it shows a peak almost identical to that of the YBCO wire indicating similar *ab*-plane correlated pinning like SFs (consistent with *H*_irr_(*T*) in Fig. 5c). However, a much broader maximum is found for |*θ*| < 80°. This is the consequence of the higher density of TBs coupled with the presence of strong random pinning from BZO NPs as shown in the TEM of Fig. 1b,c, and the schematics of Fig. 3. The NPs are responsible for the increases in the wider angular range and the TBs are more effective near the *c* axis. The broad dome-shape like increase in μ_0_*H*_irr_(*θ*)–*ε*(*θ*)μ_0_*H*_irr_ due to the NPs resembles the increases found in *J*_c_ in samples with nanosized inclusions, both in YBCO and Fe-based superconductors^10^,^26^,^27^,^28^,^29^. The reason for this dome shape (or inverse anisotropy) is found in the fact that while NPs are randomly dispersed, they are not point defects, thus do not follow the electronic mass anisotropy scaling as point defects do^21^. Recently Mishev *et al.* proposed a model to explain this effect based on the relative size of the vortex core compared to the inclusion size^29^.

The leveling off observed in *H*_irr_(*θ*) due to nanoparticles translates into an smaller ratio of *H*_irr_(||*ab*)/*H*_irr_(||*c*), with values of 2.9 for +3BZO as compared to 5.0 for YBCO. The decrease in the *H*_irr_ ratio (also found in *J*_c_) has been analyzed using an ‘effective anisotropy” (*γ*_eff_) both for of *J*_c_(*θ*) and *H*_irr_(*θ*)^6^,^7^,^30^,^31^. We attempted to scale the *H*_irr_(*θ*) data of our BZO NPs doped wires using *γ*_eff_. Using intermediate angles we obtain *γ*_eff_ ~ 2, but the scaling is not successful, as the data do not collapse onto a single curve as shown in the literature^6^,^7^,^30^,^31^. It is worth mentioning that although in principle the samples in references 6,7,14 are also MOD with NPs, there are some differences in the microstructure with the wires in this study. Specifically, with the addition of BZO our samples have a higher density of twin boundaries, but the opposite is found in ref. 14.

As shown in Fig. 5c, overall the +3BZO wire shows an extremely high μ_0_*H*_irr_ at 65 K, at 30 T (**H**||45°) and 24 T (**H**||*c*) and at 50 K, 58 T (**H**||45°) and 45 T (**H**||*c*), values which to our knowledge are the highest reported for practical HTS wires^31^ as well as for thin films on single-crystal substrates^32^. Recently, very high μ_0_*H*_irr_ = 14.8 and 15.8 T at 77 K (**H**||*c*) were observed in a heavily BZO nanorod-doped (Y,Gd)BCO wire grown by metal organic chemical vapor deposition^33^,^34^ and BaHfO_3_ doped PLD GdBCO wire^35^; these methods could also translate to great improvements of *H*_irr_ at high fields. This indicates that a high density of NPs/columns is beneficial not only for MOD prepared materials but is a general trend for REBCO wires.

## Discussion

**Figure 6:**
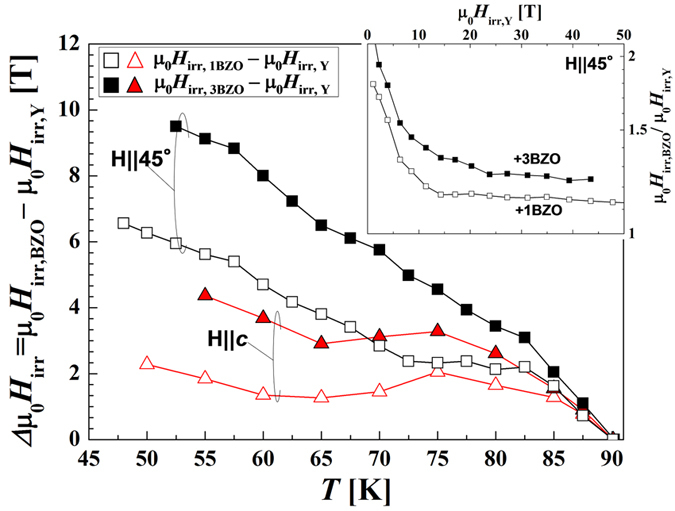
Temperature dependence of *H*_irr_ enhancement. Temperature dependence of Δμ_0_*H*_irr_ = μ_0_*H*_irr,BZO_−μ_0_*H*_irr,Y_ at **H**||*c* and **H**||45° for +1BZO and +3BZO wires. The inset shows the ratio of μ_0_*H*_irr,BZO_/μ_0_*H*_irr,Y_ for + 1 and + 3BZO for **H**||45°.

In order to pinpoint *H*-*T* regions where the BZO additions are more effective, in Fig. 6 we plot the *H*_irr_ enhancement Δμ_0_*H*_irr_ = μ_0_*H*_irr,BZO_−μ_0_*H*_irr,Y_ for different concentrations of BZO NPs. This also set the bases to determine possible *H-T* saturation regions and if so, the dependence with magnetic field orientation. The improvement, Δμ_0_*H*_irr,_ is bigger for higher BZO NP densities. We also find that Δ*H*_irr_ shows similar temperature dependences for samples with +1BZO, +2BZO (not shown) and +3BZO for all orientations measured. At **H**||45°, Δμ_0_*H*_irr_ curves have a greater increase with respect to **H**||*c* for both concentrations, consistently with Fig. 5c, showing no signs of saturation up to the highest magnetic field measured.

The lack of saturation is also apparent in the inset of Fig. 6, which shows the ratio μ_0_*H*_irr,BZO_/μ_0_*H*_irr,Y_ for the **H**||45° orientation for two different concentrations of BZO NPs, 1% and 3%. The enhancement factor is largest at low fields and initially decays with *H*, but then becomes constant up the highest accessible fields. In particular, *H*_irr_ for +3BZO is about 25% higher than for YBCO up to ~45 T.

Also observed in Fig. 6 is a steeper growth in *H*_irr_ at *T* > 80 K for both +1BZO and +3BZO wires. This temperature corresponds to μ_0_*H* ∼ 5 T, where the inter-vortex distance is *a*_f_ ∼ 22 nm, which is close to the average BZO NP spacing (*d*_BZO_ ∼ 30 nm) observed in TEM images. This suggests that NPs are most effective in increasing μ_0_*H*_irr_ (at **H**||45°) when the intervortex spacing is similar the distance between NPs. This behavior is analogous to the *J*_c_ enhancement we observed as function of field for different NP densities in ref. 36.

The fact that improvements are smaller for **H**||*c* compared to **H**||45°, is partly due to the fact that *H*_irr_ is already higher due to the presence of correlated defects (insert Fig. 5d). After an initial increase of *H*_irr_, Δμ_0_*H*_irr_ flattens around 75 K and appears to increase again for *T* < 60 K. The initial increase can be related to the higher density of NPs and/or of TBs, with the latter being 2.3 times more numerous in samples with BZO than in standard YBCO. However, the increase at lower temperatures is most likely related solely to the presence of nanoparticles, given the similarity of the behavior found at **H**||45°.

The lack of saturation in the increase of *H*_irr_ is remarkable. For +3BZO at **H**||45° we have achieved a 25% enhancement up to the highest fields measured, with no sign of decrease. This corresponds to an absolute increase over 9 T for μ_0_*H*_irr_ ~ 50 T, implying an increase of ~4–6 K in operational temperature (depending on the orientation). This enhancement is particularly significant knowing that at μ_0_*H* ∼ 60 T the inter-vortex spacing is at least 7 times smaller than the average distance between nanoparticles. These findings indicate that further improvement of *H*_irr_ in practical REBCO wires is still possible, thus pushing the technically relevant region up to even higher magnetic fields.

In summary, we find that up to the highest BZO NPs density we tested, *H*_irr_ can be increased further, with no indication of saturation up to 60 T. This allows us to report extremely high μ_0_*H*_irr_, the highest values reported so far for REBCO films and wires. Our results suggest that higher densities of NPs will increase *H*_irr_ even more, and that the high-field limit is far from being reached in nanoengineered REBCO wires. These results are further proof that REBCO wire is an enabling technology for several high field applications.

## Methods

HTS wires based on epitaxial REBCO nanocomposite films of standard YBCO and (Y,Gd)BCO + BZO were grown from metal organic solutions including Y-, Gd-, and Ba-trifluoroacetates and Cu-naphthenate with the cation ratio of 0.77 : 0.23 : 1.5 : 3 on ion-beam-assist deposited metal templates^37^. We added Zr-naphthenate into the (Y, Gd)BCO solutions; the content of BZO was 1–3wt%, and the concentration of starting solution was 1.2 mol/L. The total thickness of the superconductive layer for all samples was 0.5 μm, which was confirmed by cross sectional transmission electron microscopy (TEM). The details of the calcinations and conversion steps have been published elsewhere^17^.

Films were patterned using photolithography and ion milling into bridges of ∼50 μm width. The crystalline quality was examined by x-ray diffraction (XRD). The resistivity measurements, *ρ* vs. *H*, were realized in pulsed fields up to 60 T at the Pulsed Field Facility of the National High Magnetic Field Laboratory at Los Alamos National Laboratory. The four-probe technique was applied in AC mode with a low current corresponding to 400 A/cm^2^ and a frequency of 100 kHz. Pulsed field measurements were performed at fixed orientations (*θ*), and the angular orientation was changed using a mechanical rotator. Additional *ρ* vs. *T* measurements were performed in a Quantum Design PPMS with a superconducting magnet (DC magnetic field) generating fields μ_0_**H** up to 15 T. For the transport measurements the current was always perpendicular to **H**. Criteria of 0.9*ρ*_n_ and 0.01*ρ*_n_, where *ρ*_n_ is the normal state resistivity, were used to define *H*_c2_ and *H*_irr_, respectively. Both planar and cross sectional TEM images were taken to evaluate the microstructure of the film.

The microstructures and elemental concentration mappings of the films were analyzed by transmission electron microscopy (TEM) and energy-dispersive x-ray spectroscopy (EDS), respectively.

## Additional Information

**How to cite this article**: Miura, M. *et al.* Upward shift of the vortex solid phase in high-temperature-superconducting wires through high density nanoparticle addition. *Sci. Rep.* **6**, 20436; doi: 10.1038/srep20436 (2016).

## Figures and Tables

**Table 1:** Structural and superconducting properties.

Sample	YBCO	(Y,Gd)BCO	+1BZO	+2BZO	+3BZO
*δω* (deg.)	1.07	1.10	1.05	1.08	1.11
*δϕ* (deg.)	2.89	2.85	2.88	2.84	2.83
Average NP diameter (nm)	~108*	~113*	23 ± 4.8	23 ± 4.6	22 ± 4.7
Average NP density (10^21^/m^3^)	~0.1*	~0.1*	2.9 ± 0.1	7.1 ± 0.14	13.0 ± 0.13
Average TB spacing (nm)	45 ± 4.8	38.5 ± 5.2	34 ± 2.5	32.5 ± 3.2	30 ± 3.1
*T*_c_ (K)	89.9	90.2	90.3	90.4	90.4
*J*_c_^s.f.^ (77 K) (MA/cm^2^)	2.8	3.8	4.0	3.9	4.0

Sample data for the reference YBCO wire and for the (Y,Gd)BCO + BZO wires. *δω* and *δϕ* denote the full-width at half-maximum (FWHM) values of the out-of-plane rocking curves (*ω* scans) of the 005 diffraction peaks and in-plane rocking curves (*ϕ* scans) of the 103 diffraction peaks, respectively. The particle-size distribution was extracted from high-magnification planar- and cross-section views of several TEM images. The size of the BZO NPs ranged from 17 to 28 nm with a modal size of ∼23 nm. The average TB spacing was estimated from several planar view TEM images. *T*_c_ was determined using a criterion of 0.01*ρ*_*n*_.

^*^225 precipitates.
